# Hydrogen bonding in substitutionally disordered di-μ-hydroxido-bis­{aqua­tri[bromido/chlorido(1/2)]tin(IV)} acetone disolvate

**DOI:** 10.1107/S1600536808040543

**Published:** 2008-12-10

**Authors:** Ioana Barbul, Richard A. Varga, Cristian Silvestru

**Affiliations:** a Faculty of Chemistry and Chemical Engineering, Babes-Bolyai University, Arany Janos Street No. 11, RO-400028, Cluj Napoca, Romania

## Abstract

The structure of the title compound, [Sn_2_Br_1.97_Cl_4.03_(OH)_2_(H_2_O)_2_]·2C_3_H_6_O, contains two hexa­coordinated Sn atoms bridged symmetrically by two hydroxide groups, with an inversion center in the middle of the planar Sn_2_O_2_ ring, half of the mol­ecule being generated by inversion symmetry. The other sites of the distorted octa­hedral coordination geometry are occupied by halide atoms and water mol­ecules. The structure exhibits substitutional disorder of the halide atoms bonded to the Sn atom, with 0.672 (4) occupancy for Cl and 0.328 (4) for Br for each halide position. The compound crystallizes with two acetone mol­ecules, which are involved in intra- and inter­molecular O—H⋯O contacts. The water mol­ecules coordinated to the Sn atoms are also involved in O—H⋯O and O—H⋯*X* contacts, leading to a polymeric array along the *a* axis.

## Related literature

For related tin(IV) compounds, see: Barnes *et al.* (1980 ▶); Bokii & Struchkov (1971 ▶).
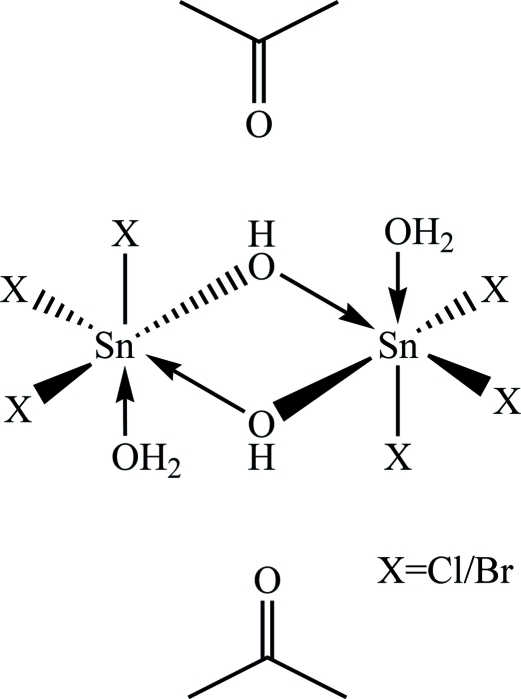


## Experimental

### 

#### Crystal data

[Sn_2_Br_1.97_Cl_4.03_(OH)_2_(H_2_O)_2_]·2C_3_H_6_O
*M* *_r_* = 723.80
Monoclinic, 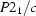

*a* = 6.9057 (13) Å
*b* = 14.029 (3) Å
*c* = 11.400 (2) Å
β = 103.195 (4)°
*V* = 1075.3 (4) Å^3^
*Z* = 2
Mo *K*α radiation
μ = 6.55 mm^−1^
*T* = 297 (2) K
0.21 × 0.20 × 0.17 mm

#### Data collection

Bruker SMART APEX CCD area-detector diffractometer
Absorption correction: multi-scan (*SADABS*; Bruker, 2000 ▶) *T*_min_ = 0.278, *T*_max_ = 0.329
5535 measured reflections
1891 independent reflections
1641 reflections with *I* > 2σ(*I*)
*R*_int_ = 0.032

#### Refinement

*R*[*F* ^2^ > 2σ(*F* ^2^)] = 0.040
*wR*(*F* ^2^) = 0.102
*S* = 1.08
1891 reflections
106 parameters
2 restraints
H atoms treated by a mixture of independent and constrained refinement
Δρ_max_ = 0.92 e Å^−3^
Δρ_min_ = −0.75 e Å^−3^

### 

Data collection: *SMART* (Bruker, 2000 ▶); cell refinement: *SAINT-Plus* (Bruker, 2001 ▶); data reduction: *SAINT-Plus*; program(s) used to solve structure: *SHELXS97* (Sheldrick, 2008 ▶); program(s) used to refine structure: *SHELXL97* (Sheldrick, 2008 ▶); molecular graphics: *DIAMOND* (Brandenburg & Putz, 2006 ▶); software used to prepare material for publication: *publCIF* (Westrip, 2009 ▶).

## Supplementary Material

Crystal structure: contains datablocks I, global. DOI: 10.1107/S1600536808040543/si2138sup1.cif

Structure factors: contains datablocks I. DOI: 10.1107/S1600536808040543/si2138Isup2.hkl

Additional supplementary materials:  crystallographic information; 3D view; checkCIF report

## Figures and Tables

**Table 1:** Hydrogen-bond geometry (Å, °)

*D*—H⋯*A*	*D*—H	H⋯*A*	*D*⋯*A*	*D*—H⋯*A*
O1—H1⋯O3	0.79 (7)	1.93 (7)	2.714 (6)	170 (7)
O2—H3⋯X3^i^	0.89 (9)	2.47 (10)	3.244 (5)	146 (8)
O2—H3⋯X1^ii^	0.89 (9)	2.88 (12)	3.483 (6)	127 (8)
O2—H2⋯O3^ii^	0.88 (5)	1.79 (5)	2.654 (7)	170 (4)

Symmetry codes: (i) 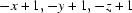; (ii) 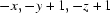.

**Fig. 1.**
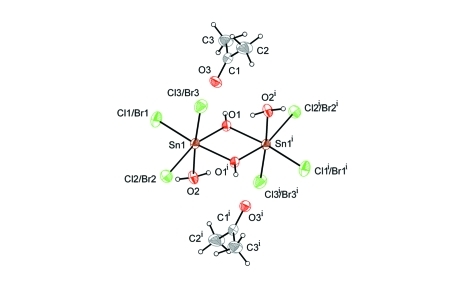
: View of the title compound showing the atom-numbering scheme at 30% probability thermal ellipsoids [symmetry code: (i) = -x + 1, -y + 1, -z + 1].

**Fig. 2.**
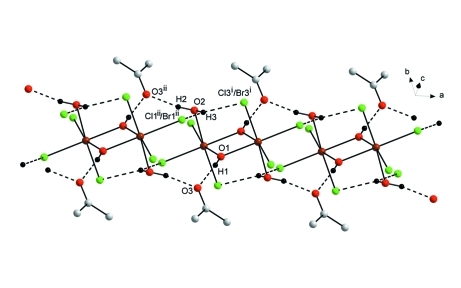
: Intra- and intermolecular interactions in the title compound (dashed lines; only H atoms involved in interactions are shown). Symmetry codes as in Table 1.

**Fig. 3.**
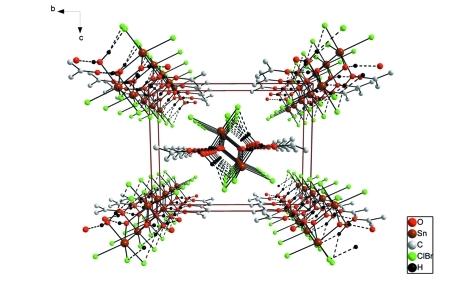
: Crystal packing of the title compound showing the supramolecular arrangement.

## supplementary crystallographic information

### Comment

The title compound forms a dimeric structure with two aquatrihalidotin(IV) fragments bridged symmetrically by two hydroxo groups (Figure 1). Half of the molecule is generated by symmetry due to the presence of the inversion center in the middle of the Sn_2_O_2_ ring. This ring is planar and describes a rhomb with the endocyclic angles at O larger than those at the Sn atoms [Sn1—O1—Sn1^i^ = 109.2 (2)°, O1—Sn1—O1^i^ = 70.8 (2)°; symmetry code: (i) = -*x* + 1, -*y* + 1, -*z* + 1]. The tin atoms are hexacoordinated with the two hydroxo, three halides and one water molecule occupying the distorted octahedral positions around the metal centre. The tin atoms are out of the best plane described by O1/O1^i^/X1/X2 (*X* = Cl/Br) with 0.174 Å towards X3.

The compound exhibits substitutional disorder of the halide atoms bonded to the Sn with 0.672 occupancy for Cl and 0.328 for Br for each halide position.

The compound crystallizes with two acetone molecules, which establish two strong hydrogen bonds, one with the hydroxo group and one with the water from a neighboring dimer (Table 1). The water molecules are also involved in hydrogen bond type interactions with halide atoms, a strong one inside the dimeric unit and one intermolecular with a halide from another dimer (Table 1). The intramolecular interactions strengthen the dimeric unit and the intermolecular ones give rise to a polymer-like supramolecular arrangement along the *a* axis (Figure 2), with no further interactions between different chains (Figure 3).

### Experimental

The title compound was obtained as a by-product after the work up of the crude reaction mixture obtained by reacting [2,6-(Me)_2_C_6_H_3_]MgBr and SnCl_4_.

### Refinement

The hydrogen atoms of the methyl groups were placed in calculated positions and were allowed to rotate but not to tip, with C—H = 0.96 Å and with *U*_iso_(H) = 1.5*U*_eq_(C). The three halide atoms were refined as substitutional disorder between chlorine and bromine, with 0.672 occupancy for Cl and 0.328 occupancy for Br for each position. Hydrogen atoms from the water molecule and hydroxyl group were found from a difference map and refined with a restrained O—H distance of 0.88 (5) Å,0.89 (9) Å and 0.79 (7) Å, with *U*_iso_(H) = (1.5, 3.0, and 1.2)*U*_eq_(O), respectively.

### Crystal data

**Table:**

[Sn_2_Br_1.97_Cl_4.03_(OH)_2_(H_2_O)_2_]·2C_3_H_6_O	*F*(000) = 680
*M**_r_* = 723.80	*D*_x_ = 2.240 Mg m^−^^3^
Monoclinic, *P*2_1_/*c*	Mo *K*α radiation, λ = 0.71073 Å
Hall symbol: -P 2ybc	Cell parameters from 1714 reflections
*a* = 6.9057 (13) Å	θ = 2.3–24.6°
*b* = 14.029 (3) Å	µ = 6.55 mm^−^^1^
*c* = 11.400 (2) Å	*T* = 297 K
β = 103.195 (4)°	Block, colourless
*V* = 1075.3 (4) Å^3^	0.21 × 0.20 × 0.17 mm
*Z* = 2	

### Data collection

**Table:**

Bruker SMART APEX CCD area-detector diffractometer	1891 independent reflections
Radiation source: fine-focus sealed tube	1641 reflections with *I* > 2σ(*I*)
graphite	*R*_int_ = 0.032
φ and ω scans	θ_max_ = 25.0°, θ_min_ = 2.3°
Absorption correction: multi-scan (*SADABS*; Bruker, 2000)	*h* = −7→8
*T*_min_ = 0.278, *T*_max_ = 0.329	*k* = −16→13
5535 measured reflections	*l* = −13→13

### Refinement

**Table:**

Refinement on *F*^2^	Primary atom site location: structure-invariant direct methods
Least-squares matrix: full	Secondary atom site location: difference Fourier map
*R*[*F*^2^ > 2σ(*F*^2^)] = 0.040	Hydrogen site location: inferred from neighbouring sites
*wR*(*F*^2^) = 0.102	H atoms treated by a mixture of independent and constrained refinement
*S* = 1.08	*w* = 1/[σ^2^(*F*_o_^2^) + (0.049*P*)^2^ + 2.7199*P*] where *P* = (*F*_o_^2^ + 2*F*_c_^2^)/3
1891 reflections	(Δ/σ)_max_ < 0.001
106 parameters	Δρ_max_ = 0.92 e Å^−^^3^
2 restraints	Δρ_min_ = −0.75 e Å^−^^3^

### Special details

**Table:**

Geometry. All e.s.d.'s (except the e.s.d. in the dihedral angle between two l.s. planes) are estimated using the full covariance matrix. The cell e.s.d.'s are taken into account individually in the estimation of e.s.d.'s in distances, angles and torsion angles; correlations between e.s.d.'s in cell parameters are only used when they are defined by crystal symmetry. An approximate (isotropic) treatment of cell e.s.d.'s is used for estimating e.s.d.'s involving l.s. planes.
Refinement. Refinement of *F*^2^ against ALL reflections. The weighted *R*-factor *wR* and goodness of fit *S* are based on *F*^2^, conventional *R*-factors *R* are based on *F*, with *F* set to zero for negative *F*^2^. The threshold expression of *F*^2^ > σ(*F*^2^) is used only for calculating *R*-factors(gt) *etc*. and is not relevant to the choice of reflections for refinement. *R*-factors based on *F*^2^ are statistically about twice as large as those based on *F*, and *R*- factors based on ALL data will be even larger.

### Fractional atomic coordinates and isotropic or equivalent isotropic displacement parameters (Å^2^)

**Table:**

	*x*	*y*	*z*	*U*_iso_*/*U*_eq_	Occ. (<1)
O2	0.2519 (8)	0.6291 (4)	0.5292 (5)	0.0490 (12)	
Sn1	0.34131 (6)	0.55296 (3)	0.38433 (4)	0.03550 (19)	
O1	0.3796 (6)	0.4390 (3)	0.5018 (4)	0.0370 (11)	
O3	0.1078 (7)	0.2974 (3)	0.4886 (5)	0.0535 (13)	
C1	0.1355 (11)	0.2115 (5)	0.4779 (6)	0.0475 (17)	
C2	0.3092 (13)	0.1783 (7)	0.4365 (9)	0.080 (3)	
H2A	0.2750	0.1715	0.3504	0.120*	
H2B	0.3517	0.1179	0.4729	0.120*	
H2C	0.4150	0.2239	0.4589	0.120*	
C3	−0.0070 (14)	0.1438 (6)	0.5094 (8)	0.073 (3)	
H3A	−0.0928	0.1768	0.5512	0.109*	
H3B	0.0640	0.0947	0.5603	0.109*	
H3C	−0.0852	0.1157	0.4372	0.109*	
Br2	0.3538 (2)	0.70915 (9)	0.29307 (11)	0.0567 (4)	0.328 (4)
Br3	0.4654 (2)	0.46498 (12)	0.23225 (13)	0.0661 (5)	0.328 (4)
Br1	−0.0046 (2)	0.51539 (12)	0.30386 (13)	0.0642 (5)	0.328 (4)
Cl2	0.3538 (2)	0.70915 (9)	0.29307 (11)	0.0567 (4)	0.672 (4)
Cl3	0.4654 (2)	0.46498 (12)	0.23225 (13)	0.0661 (5)	0.672 (4)
Cl1	−0.0046 (2)	0.51539 (12)	0.30386 (13)	0.0642 (5)	0.672 (4)
H2	0.128 (5)	0.647 (6)	0.520 (7)	0.07 (3)*	
H1	0.307 (11)	0.396 (5)	0.506 (6)	0.04 (2)*	
H3	0.282 (19)	0.587 (7)	0.589 (8)	0.15 (5)*	

### Atomic displacement parameters (Å^2^)

**Table:**

	*U*^11^	*U*^22^	*U*^33^	*U*^12^	*U*^13^	*U*^23^
O2	0.041 (3)	0.052 (3)	0.055 (3)	0.016 (2)	0.013 (2)	0.008 (3)
Sn1	0.0296 (3)	0.0384 (3)	0.0370 (3)	−0.00171 (19)	0.00458 (19)	0.00437 (19)
O1	0.026 (2)	0.034 (3)	0.048 (3)	−0.008 (2)	0.003 (2)	0.010 (2)
O3	0.042 (3)	0.033 (3)	0.086 (4)	0.000 (2)	0.015 (3)	0.001 (3)
C1	0.046 (4)	0.046 (5)	0.045 (4)	0.001 (3)	−0.002 (3)	−0.003 (3)
C2	0.070 (6)	0.073 (6)	0.103 (7)	0.003 (5)	0.030 (6)	−0.034 (6)
C3	0.092 (7)	0.045 (5)	0.077 (6)	−0.013 (5)	0.011 (5)	0.004 (4)
Br2	0.0705 (9)	0.0447 (8)	0.0525 (7)	−0.0056 (6)	0.0091 (6)	0.0170 (6)
Br3	0.0596 (9)	0.0805 (11)	0.0572 (8)	0.0018 (7)	0.0112 (7)	−0.0109 (7)
Br1	0.0400 (8)	0.0857 (11)	0.0624 (8)	−0.0056 (7)	0.0025 (6)	0.0144 (8)
Cl2	0.0705 (9)	0.0447 (8)	0.0525 (7)	−0.0056 (6)	0.0091 (6)	0.0170 (6)
Cl3	0.0596 (9)	0.0805 (11)	0.0572 (8)	0.0018 (7)	0.0112 (7)	−0.0109 (7)
Cl1	0.0400 (8)	0.0857 (11)	0.0624 (8)	−0.0056 (7)	0.0025 (6)	0.0144 (8)

### Geometric parameters (Å, °)

**Table:**

O2—Sn1	2.171 (5)	O3—C1	1.230 (8)
O2—H2	0.88 (5)	C1—C2	1.462 (11)
O2—H3	0.89 (9)	C1—C3	1.470 (11)
Sn1—O1	2.064 (4)	C2—H2A	0.9600
Sn1—O1^i^	2.066 (4)	C2—H2B	0.9600
Sn1—Br1	2.4138 (14)	C2—H2C	0.9600
Sn1—Br2	2.4357 (13)	C3—H3A	0.9600
Sn1—Br3	2.4376 (16)	C3—H3B	0.9600
O1—Sn1^i^	2.066 (4)	C3—H3C	0.9600
O1—H1	0.79 (7)		
			
Sn1—O2—H2	119 (6)	Sn1—O1—Sn1^i^	109.2 (2)
Sn1—O2—H3	102 (9)	Sn1—O1—H1	130 (5)
H2—O2—H3	110 (10)	Sn1^i^—O1—H1	121 (5)
O1—Sn1—O1^i^	70.8 (2)	O3—C1—C2	120.0 (7)
O1—Sn1—O2	84.5 (2)	O3—C1—C3	118.8 (7)
O1^i^—Sn1—O2	83.21 (19)	C2—C1—C3	121.2 (8)
O1—Sn1—Br1	92.67 (13)	C1—C2—H2A	109.5
O1^i^—Sn1—Br1	162.00 (13)	C1—C2—H2B	109.5
O2—Sn1—Br1	88.16 (15)	H2A—C2—H2B	109.5
O1—Sn1—Br2	164.26 (14)	C1—C2—H2C	109.5
O1^i^—Sn1—Br2	95.74 (13)	H2A—C2—H2C	109.5
O2—Sn1—Br2	85.79 (15)	H2B—C2—H2C	109.5
Br1—Sn1—Br2	99.36 (5)	C1—C3—H3A	109.5
O1—Sn1—Br3	93.18 (14)	C1—C3—H3B	109.5
O1^i^—Sn1—Br3	92.62 (14)	H3A—C3—H3B	109.5
O2—Sn1—Br3	175.70 (14)	C1—C3—H3C	109.5
Br1—Sn1—Br3	95.57 (6)	H3A—C3—H3C	109.5
Br2—Sn1—Br3	95.70 (5)	H3B—C3—H3C	109.5
			
O1^i^—Sn1—O1—Sn1^i^	0.0	Br2—Sn1—O1—Sn1^i^	−32.6 (6)
O2—Sn1—O1—Sn1^i^	−84.7 (2)	Br3—Sn1—O1—Sn1^i^	91.67 (19)
Br1—Sn1—O1—Sn1^i^	−172.60 (19)		

Symmetry codes: (i) −*x*+1, −*y*+1, −*z*+1.

### Hydrogen-bond geometry (Å, °)

**Table:**

*D*—H···*A*	*D*—H	H···*A*	*D*···*A*	*D*—H···*A*
O1—H1···O3	0.79 (7)	1.93 (7)	2.714 (6)	170 (7)
O2—H3···X3^i^	0.89 (9)	2.47 (10)	3.244 (5)	146 (8)
O2—H3···X1^ii^	0.89 (9)	2.88 (12)	3.483 (6)	127 (8)
O2—H2···O3^ii^	0.88 (5)	1.79 (5)	2.654 (7)	170 (4)

Symmetry codes: (i) −*x*+1, −*y*+1, −*z*+1; (ii) −*x*, −*y*+1, −*z*+1.
